# Heterodyne mixing of millimetre electromagnetic waves and sub-THz sound in a semiconductor device

**DOI:** 10.1038/srep30396

**Published:** 2016-08-01

**Authors:** Sarah L. Heywood, Boris A. Glavin, Ryan P. Beardsley, Andrey V. Akimov, Michael W. Carr, James Norman, Philip C. Norton, Brian Prime, Nigel Priestley, Anthony J. Kent

**Affiliations:** 1 School of Physics and Astronomy, University of Nottingham, University Park, Nottingham NG7 2RD UK; 2 Department of Theoretical Physics, V E Lashkaryov Institute of Semiconductor Physics, Pr. Nauki 41, Kyiv, Ukraine; 3 e2v Microwave Technology Centre, Firth Road Business Centre, Firth Road, Lincoln LN6 7AA UK

## Abstract

We demonstrate heterodyne mixing of a 94 GHz millimetre wave photonic signal, supplied by a Gunn diode oscillator, with coherent acoustic waves of frequency ~100 GHz, generated by pulsed laser excitation of a semiconductor surface. The mixing takes place in a millimetre wave Schottky diode, and the intermediate frequency electrical signal is in the 1–12 GHz range. The mixing process preserves all the spectral content in the acoustic signal that falls within the intermediate frequency bandwidth. Therefore this technique may find application in high-frequency acoustic spectroscopy measurements, exploiting the nanometre wavelength of sub-THz sound. The result also points the way to exploiting acoustoelectric effects in photonic devices working at sub-THz and THz frequencies, which could provide functionalities at these frequencies, e.g. acoustic wave filtering, that are currently in widespread use at lower (GHz) frequencies.

In typical solid materials, sub-terahertz (THz) coherent acoustic waves have wavelengths in the nanometre range. Optical techniques for the generation and detection of such nanoacoustic waves, based on the use of ultrafast (femto- and picosecond) pulsed lasers^1^, have now reached a level of maturity whereby acoustic wavepackets with frequencies in the range 50 GHz to several THz with strain amplitudes of up to 10^−3^ are routinely available, for a recent review see ref. 2. This approach extends established ultrasonic methods of non-destructive probing to address the much shorter length scales relevant to modern nanoscience and nanotechnology developments, for example imaging of objects with nanometre resolution^3^. Hence, a strong international research effort is currently aimed at using nanoacoustic waves to probe, and even to alter dynamically, the physical properties of nanostructured materials and devices. Such nanoacoustic waves can, for example, be used for the ultrafast modulation of the electronic states in semiconductor nanodevices, which allows control over their optical^4^,^5^ and electrical transport^6^,^7^,^8^,^9^ properties.

However, despite their successes, the laser-based nanoacoustics techniques suffer from a serious drawback, which has limited their wider use across scientific disciplines and in industry. This is the need for large and costly ultrafast laser systems and the attendant complex optical pump-probe setups requiring highly trained operators to set up and maintain. Thus nanoacoustics measurements have so far been largely restricted to specialist research labs possessing the necessary hardware and skills.

A development that could lead to a more widespread adoption of nanoacoustics would be methods of generation and detection of the nanoacoustic waves, similar to the methods already in widespread use at lower acoustic frequencies. Even a combination of optical generation, e.g. using a picosecond pulsed laser diode or fibre laser, and acoustoelectric detection would still offer the advantage of considerable simplification of the optical setup.

A number of alternatives to optical probing of nanoacoustic waves have been demonstrated. These include: superconducting bolometers^10^ and semiconductor electronic devices such as p-n, Schottky and resonant tunnelling diodes and superlattices^6^,^7^,^8^,^9^,^11^, all of which generate electrical signals in response to incident nanoacoustic wave packets. However, unlike the optical-based detection methods, most of these devices detect the signal by rectification and offer little or no spectral resolution. The Schottky diode is potentially a fast enough device to allow spectral resolution up to about one terahertz, but measurement of the signals is limited to ~10 GHz by the cabling and signal acquisition systems. It has also been demonstrated that a high frequency acoustic wave can be converted directly to an electromagnetic wave using a piezoelectric structure^12^. This method could provide the required spectral resolution, but again, specialized techniques, e.g. electro-optic sampling, are required to detect the electromagnetic signal produced.

In view of these considerations, a photonic method for acoustic wave detection, which uses neither sophisticated THz electronics nor ultrafast lasers, would be desirable. In this work we explore such an approach exploiting the high internal speed of response and non-linear electrical characteristic of a Schottky diode to mix the acoustically-induced signal with a millimetre wave photonic signal injected from a local oscillator. The mixing produces intermediate frequency electrical signals in the GHz range, allowing measurements in real-time using digitizing oscilloscopes. The important point here is that the spectral information in the acoustic signal is preserved in the mixing process; it is just converted to a lower frequency band where it can be digitized and processed more easily. Owing to rapid development in mobile, satellite and data communications technology, electrical measurement equipment, e.g. digital oscilloscopes and spectrum analysers, for the ~10 GHz frequency range are widely available and at a reasonable cost.

Here we investigate heterodyne mixing of coherent acoustic waves with electromagnetic waves in a millimetre wave Schottky diode. The scheme of the experiment is shown in Fig. 1: acoustic waves are injected into the diode via the semiconductor substrate, and the millimetre waves coupled to the diode electrically. The intermediate (difference) frequency signal output by the diode is detected using a wide-bandwidth digital oscilloscope. In the work described in this article, the acoustic wave is generated by femtosecond laser excitation of the semiconductor, but it would not be restricted to this method of generation. For example, the acoustic wave could be emitted by various vibrating nano-objects, such as semiconductor superlattices, and the heterodyne setup would allow the precise measurement of the spectrum of the emitted acoustic wave.

## Materials and Methods

**Figure 1:**
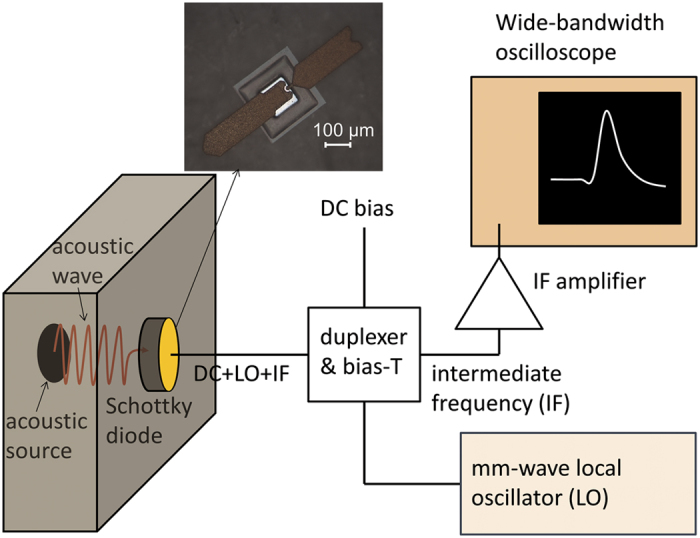
Scheme for mixing the frequencies of an acoustic wave and a mm-wave electromagnetic source. The mixing device is a mm-wave beam lead Schottky diode shown in the photograph. The acoustic source used in this work is the back surface of the semiconductor (GaAs) substrate, excited by a train of femtosecond laser pulses. However, any other source of sub-THz acoustic waves, e.g. vibrating nano objects, could be used and their spectrum studied.

The heterodyne mixing experiment using a Schottky diode as the mixer device is shown schematically in Fig. 1. The millimetre wave local oscillator (LO) signal is provided by a Gunn diode oscillator running at 94 GHz. This is mixed with the quasi-monochromatic acoustic signal in the frequency range 84–106 GHz produced by optically pumping the surface of the GaAs substrate, opposite to the Schottky diode, with a train of femtosecond optical pulses. The optical pulse train is produced by a femtosecond laser with an external Fabry-Perot cavity. The acoustic frequency of interest is the second harmonic of the frequency of the pulse train, *f*_0_, produced by the Fabry-Perot cavity, and is tuned by changing the cavity spacing.

We used an n-type GaAs Beam Lead millimetre wave Schottky diode manufactured by e2v plc. The Schottky contact area is 5 × 5 microns and the electrical connection brought out via an air bridge (beam) lead to minimise capacitance and dielectric losses at high frequency. Such diodes are normally used in millimetre-wave detection applications up to ~125 GHz. The diode was mounted on a millimetre wave circuit board, which was designed to allow coupling to the diode of the 94 GHz local oscillator (LO) signal from an e2v Gunn diode oscillator module via a waveguide, and extraction of the 0–12.5 GHz intermediate frequency (IF) signal via a coaxial (SMA) lead. A bias-T was inserted in the IF lead so that a direct current (DC) bias could be applied to the diode if required.

Acoustic waves were generated by direct optical excitation of the back of the ≈11 micron-thick GaAs substrate, on which the Schottky diode is fabricated, by femtosecond-duration pulses of 800 nm wavelength produced by an amplified Ti:Sapphire laser with a pulse repetition rate of 5 KHz. Absorption of such a light pulse at the surface of the GaAs substrate is known to give rise to the generation of an acoustic (strain) pulse of picoseconds duration. Owing to the sharp interface between the air, in which the laser pulse is propagating, and the surface of the GaAs, this strain pulse can have frequency components extending up to ~100 GHz^13^. Mixing of such an acoustic signal with the local oscillator signal would give rise to a very broad spectrum IF signal, which would be difficult to resolve from the noise background. So, for these mixing experiments, we required an acoustic signal with a (quasi-) monochromatic spectrum. It had been shown that such an acoustic signal can be produced using a train of closely separated in time strain pulses^14^. The train of strain pulses is generated by a train of femtosecond optical pulses produced using a “pulse shaper”. In this work, we used a simple pulse shaping device: a Fabry-Perot cavity inserted in the laser beam. For an input to the cavity of a single femtosecond light pulse, the output consists of a train of femtosecond light pulses, exponentially decaying in intensity, with the frequency *f*_0_ = *c*/2*d*, where *d* is the spacing of the cavity mirrors; for example, for *d* = 3 mm *f*_0_ = 50 GHz. This train of light pulses was focussed to a spot of diameter ~100 μm on the GaAs surface opposite the Schottky device, producing an acoustic wavepacket consisting of a train of picosecond-duration strain pulses. This acoustic wavepacket has significant spectral components at the frequencies: *f*_0_; 2*f*_0_, …, *nf*_0_, where *nf*_0_ < ~ 100 GHz, which is the highest frequency component in a picosecond acoustic pulse generated by a single light pulse. By adjusting *d*, it was possible to tune *f*_0_ over the range ~10–100 GHz. In the mixing experiments we used the second harmonic of *f*_0_ ~ 50 GHz because it is more efficient for generating a quasi-monochromatic acoustic wave at ~100 GHz from such a pulse train than using the fundamental of *f*_0_ ~ 100 GHz^11^. Further details on the generation and characterisation of the acoustic wave packet can be found in the Supplementary Methods, where it is also shown that, at room temperature, the 100 GHz acoustic wave is able to propagate through the thin GaAs substrate and reach the other side.

The IF output from the Schottky diode was amplified using broadband (0.3 MHz–14 GHz) microwave amplifier modules and fed into a real-time digitising oscilloscope (Tektronix DPO71254), which had an analogue bandwidth of 12.5 GHz and sampling rate of 50 GSa/s. The oscilloscope was equipped with software to calculate the Fourier power spectrum of the displayed time domain signal.

## Results and Discussion

We first consider the behaviour of the Schottky detector diode when exposed to the millimetre wave and acoustic wave excitations separately, before moving on to consider the response when they are applied together.

**Figure 2:**
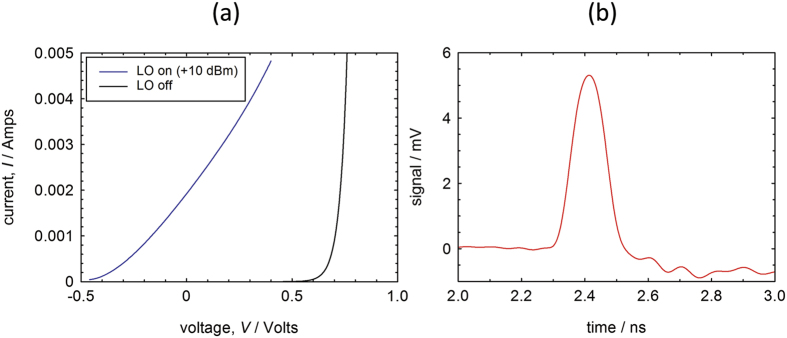
Schottky diode behavior. (**a**) *I–V* characteristics with and without the LO signal applied. *V* is the value of the applied DC bias; (**b**) Response of Schottky diode to incident acoustic burst (with the 94 GHz LO signal turned off.

Figure 2(a) shows the DC current-voltage characteristics of the detector diode without and with the 94 GHz microwaves (power level = + 10 dBm) applied. The increase in the forward current for biases below the DC forward turn-on voltage of the diode (≈0.6 V) when millimetre waves are applied shows that the diode is rectifying the LO signal.

The diode’s response to the acoustic wavepacket, with the millimetre waves turned off and with an applied DC forward voltage bias of 0.84 V, is shown in Fig. 2(b). In this case, the Fabry-Perot cavity was set to produce pulses at a frequency of about 50 GHz. The zero of time corresponds to the laser pulses hitting the back surface of the GaAs substrate and the longitudinal acoustic waves reach the Schottky junction 2.3 ns later, corresponding to the time of flight across a substrate thickness of ≈11 microns. A transient increase in the diode current is observed. Due to the limited bandwidth of the microwave amplifier modules and oscilloscope, it is impossible to resolve the acoustic component at 50 GHz. However, the overall duration of the signal is consistent with ~10 individual acoustic pulses being time integrated by the detection system.

The physical mechanisms of the acoustoelectric response of a Schottky diode were considered in our previous work^8^ and will not be discussed in detail here. In short: under forward bias, the main effect of the incident acoustic packet is to induce charge redistribution in the spatially non uniform portions of the structure, first of all at the edge of the depletion region of semiconductor and metal-semiconductor interface. This redistribution is due to electron screening of the deformation potential induced by the strain pulse and it gives rise to the displacement current while the acoustic wave passes through them.

Turning the 94 GHz signal on reduced slightly the overall strength of the measured acoustic response, probably due to the shift of the diode’s operating point by the rectified 94 GHz signal. However, it was not possible to see evidence of mixing products in the temporal trace at any cavity spacing. This is not surprising since the Gunn diode millimetre wave oscillator and the laser were not phase-locked, and so the random phase fluctuations of the mixing products will have resulted in the signal being lost when signal averaging in the time domain.

**Figure 3:**
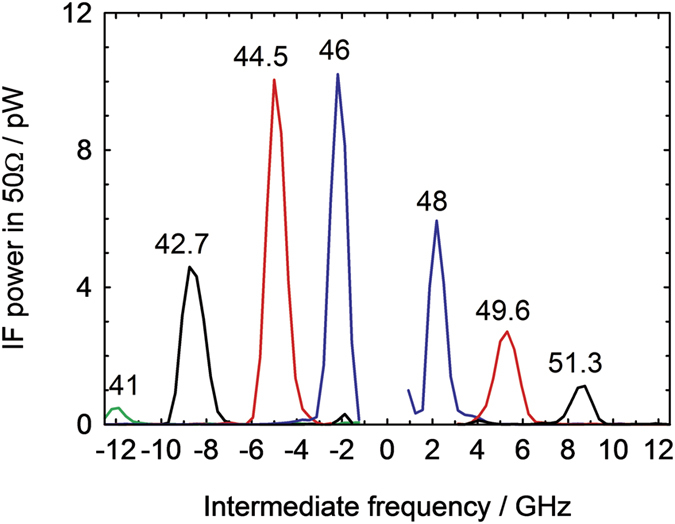
IF power as a function of the intermediate frequency. For clarity of presentation, negative (positive) frequencies correspond to 2*f*_0_ being less (greater) than the 94 GHz LO frequency, and the values of *f*_0_ (in GHz) determined by the Fabry-Perot cavity spacing are marked on the corresponding peaks. The resolution bandwidth of the oscilloscope spectrum analysis was 500 MHz, corresponding to a 2 ns-duration signal capture window.

Instead of signal averaging in the temporal regime, the Fourier power spectra of the individual temporal traces, which repeat at the frequency of the laser (=5 kHz), were averaged and the result shown in Fig. 3. The range of *f*_0_ tuned by the Fabry-Perot cavity spacing was 41–52 GHz and we detect a signal corresponding to the difference (intermediate) frequency *f*_IF_ between the second harmonic 2*f*_0_ and the 94 GHz LO. For the sake of clarity, the results for positive (negative) difference 2*f*_0_ – 94 GHz are given for positive (negative) frequency range. A peak is clearly seen at frequency *f*_IF_ = 2*f*_0_ – 94 GHz, which moves in frequency as the Fabry-Perot cavity is tuned (a Supplementary Video of this result is available).

The Gunn diode LO has a specified phase noise of between −75 and −80 dBc/Hz at 100 KHz from the centre frequency, and this corresponds to a spectral width of the 94 GHz signal of less than 20 MHz. Therefore the spectral width (full width at half maximum) of the IF signal, ~1.5 GHz, is expected to be the same as the spectral width of the 2*f*_0_ component of the acoustic packet.

**Figure 4:**
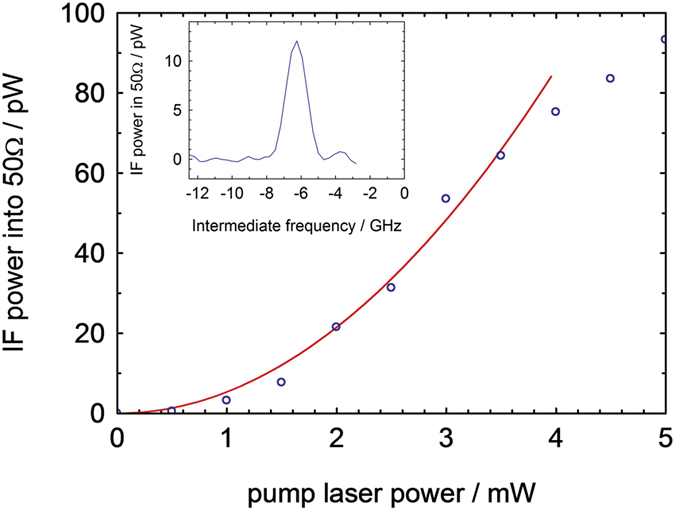
Dependence of peak IF power, at an intermediate frequency (2*f*_0_ – 94 GHz) = −6.2 GHz, on pump laser power. The red curve indicates square-law behavior. The inset shows the IF spectrum corresponding to a pump power of 1.5 mW.

Figure 4 shows the intermediate frequency (IF) signal power as a function of the pump laser power. The dependence is approximately square-law up to a pump power of 3.5 mW, corresponding to a pump pulse energy density of ~7 mJ/cm^2^, after which the signal starts to deviate from the square law behaviour. We estimate that ~7 mJ/cm^2^ corresponds to a strain amplitude of the acoustic wave of order 10^−4^, which corresponds to a peak power of the acoustic pulse incident on the Schottky contact of about 75 μW.

The results shown in Fig. 3 and the video in the supplemental material provide clear experimental evidence that the Schottky diode is mixing the frequencies of the acoustic and millimetre wave signals, thus providing heterodyne detection of the acoustic wavepacket with spectral resolution. Using the results for the attenuation of 56 GHz longitudinal phonons in GaAs^15^, we can estimate that the mean free path of 100 GHz phonons in GaAs at room temperature is of the order the substrate thickness. Therefore, when the cavity spacing is set such that *f*_0_ ~ 50 GHz, we can expect an IF signal due to direct mixing of the 2*f*_0_ component in the acoustic wavepacket with the electrical signal at *f*_*LO*_ (=94 GHz). We now discuss the mixing process in more detail.

As in the case of mixing of pure electrical signals, mixing of acoustic and electric ones in a Schottky diode is due to the nonlinear properties of the device. Such nonlinearity is, first, due to its nonlinear current-voltage characteristics, and, second, due to the bias dependence of the junction capacitance. Under the forward DC bias, the nonlinearity due to variation of the diode capacitance is less efficient than that of the current-voltage characteristic, and we restrict ourselves by the latter effect.

**Figure 5:**
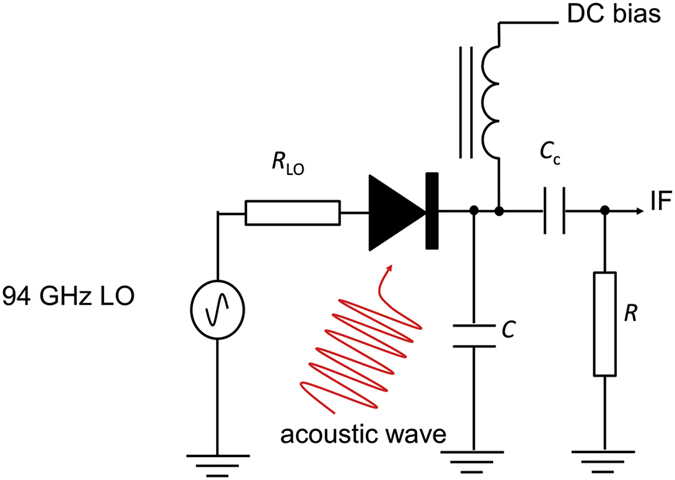
Equivalent electrical circuit of Schottky diode single-ended mixer. The capacitor *C* models a filter on the microwave circuit board that the diode is mounted on, which behaves as a short circuit at high frequency (about that of LO) while being an effective open circuit at the, much lower, intermediate frequency and at DC. The coupling capacitance *Cc* has a low impedance at the IF frequency. The source impedance of the LO is *R*_LO_ and the load impedance presented by the oscilloscope is *R*.

The circuit under consideration is shown in Fig. 5. The capacitor *C* models a filter, which behaves as a short circuit at high frequency (about that of LO) while being an effective open circuit at the, much lower, IF and at DC. The coupling capacitance *C*_*c*_ has a low impedance at the IF. For comparison with the experimental measurements, we calculate the spectrum of the power *P* dissipated at the resistor *R*, averaged over the initial phase of the LO signal at the instant when the fs laser launches the acoustic wave propagation. The details of the theoretical calculations, for the case of a high Q-factor external Fabry-Perot cavity corresponding to the experimental situation, are provided in the Supplementary Methods. We consider the case of high amplitude LO injection which is sufficient to switch the Schottky diode fully on and off on alternate half-cycles, and obtain


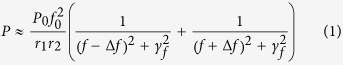


where 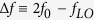, 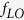 is the LO frequency; and 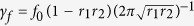 with *r*_1,2_ being the reflection coefficients of the mirrors of the Fabry-Perot cavity. *P*_0_ is the spectrum of the power dissipated in *R* due to the strain wave generated by the first optical pulse to exit the Fabry-Perot cavity. We see the main result of the calculations is that *P* has Lorentz shape as a function of frequency *f*.

**Figure 6:**
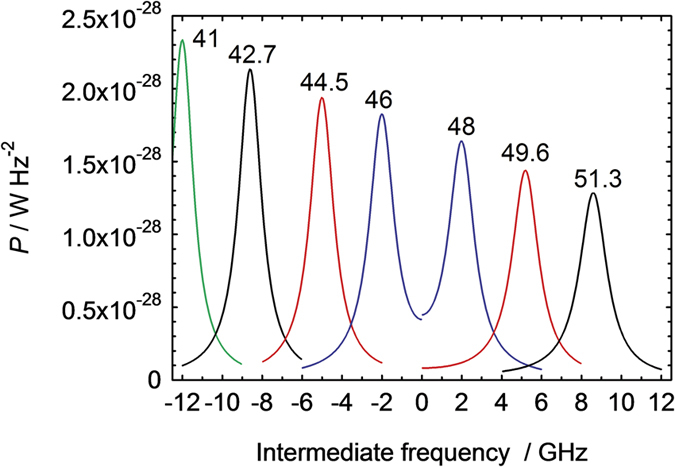
Theoretical calculation of the IF spectrum as function of the intermediate frequency. For clarity of presentation, negative (positive) frequencies correspond to 2*f*_0_ being less (greater) than the 94 GHz LO frequency, and the values of *f*_0_ determined by the Fabry-Perot cavity spacing are marked on the corresponding peaks.

In Fig. 6 we show the results obtained using Eq. (1), with *P*_0_ as given in Eq. (7) in the supplemental material. The theoretical analysis accounts very well for two of the main features of the experiment: the centre frequency of the peaks, and their full width at half maximum, ≈1.5 GHz. Comparison of the magnitude of the peaks is difficult due to uncertainties in the value of the deformation potential electron-phonon coupling, coefficient, the magnitude and shape of the strain waves and any filtering effects due to the electrical circuit and phonon scattering in the GaAs substrate. The effect of the filtering effects are clear when comparing the relative magnitude of the peaks: in the experiment, the *f*_0_ = 41 GHz peak is strongly suppressed relative to the others, which is probably due to the IF being close to the upper frequency cutoff of the oscilloscope and cables. However, considering the peak at *f*_0_ = 44.5 GHz, well clear of any IF bandwidth limits and taking into account 500 MHz resolution bandwidth of the oscilloscope spectrum analyser, the experiment gives *P* = 10 pW/(500 × 10^6^)^2^ = 4 × 10^−29^ W Hz^−2^. This is in order of magnitude agreement with the theoretical result. However, the tails of the Lorentz-like peaks are not seen in the experiment. This is probably due to their being obscured within the broadband noise background from the power spectrum averaging, which was subtracted from the signal shown in Fig. 3.

According to eq. (7) in Supplementary Methods, *P*_0_ and hence the peak IF power should be proportional to the square of the acoustic (strain) wave amplitude and hence the square of the pump laser power. The behavior up to a pump power of 3.5 mW observed in Fig. 4 is, therefore, fully consistent with the above analysis. The deviation from the square-law behavior at higher pump power (>3.5 mW) is probably due to nonlinear effects coming into play in the strain generation process. In the case of high-amplitude LO injection considered, *P*_0_ is independent on LO power.

## Conclusion

We have demonstrated heterodyne mixing of acoustic and electromagnetic signals at frequencies in the 100 GHz-range using a millimeter wave Schottky diode. Knowing the microwave local oscillator frequency and using a spectrum analyser based on a 12.5 GHz digitizing oscilloscope, we were able to determine the spectrum of the ~100 GHz acoustic signals. Apart from the potential applications in acoustic phonon spectroscopy of nanostructures, this effect could be exploited for the acoustic manipulation of terahertz electromagnetic waves. Acoustoelectric effects are already widely used at lower microwave (~ few GHz) frequencies, and this development opens the possibility of applying acoustoelectric effects at up to THz frequencies. The mixing demonstrated here could be used for detection, frequency conversion and modulation of THz signals. For example, a millimeter wave receiver using an on-chip sound laser (saser)^16^ as a stable acoustic local oscillator.

## Additional Information

**How to cite this article**: Heywood, S. L. *et al*. Heterodyne mixing of millimetre electromagnetic waves and sub-THz sound in a semiconductor device. *Sci. Rep.* **6**, 30396; doi: 10.1038/srep30396 (2016).

## Supplementary Material

Supplementary Information

Supplementary Information
