# Supplementary material for: Heterodyne mixing of millimetre electromagnetic waves and sub-THz sound in a semiconductor device
Source: Sci Rep. 2016 Aug 1;6:30396. doi: 10.1038/srep30396 (PMC4967867; doi:10.1038/srep30396)
Supplement: Supplementary Information [file srep30396-s1.doc]

**Heterodyne mixing of millimetre electromagnetic waves and sub-THz sound in a semiconductor device**

Sarah L Heywood, Boris A Glavin, Ryan P Beardsley, Andrey V Akimov

Michael W Carr, James Norman, Philip C Norton, Brian Prime, Nigel Priestley and Anthony J Kent

**Supplementary Methods**

**A. Generation and characterization of the acoustic signal**

The Fabry-Perot cavity pulse shaper device is shown schematically in Fig. S1. A light pulse of duration ≈ 60 fs, wavelength 800 nm and peak energy ~1 mJ from a 5 kHz amplified Ti:Sapphire laser was input to the cavity. The corresponding cavity output consists of a train of femtosecond light pulses of exponentially-decaying intensity and temporal separation *t*_0_ = 2*d*/*c*, where *d* is the spacing of the cavity mirrors and *c* is the speed of light. When this train of light pulses is incident on the GaAs sample, the resulting acoustic spectrum will have a components centred at frequency *f*_0_ = *c*/2*d* and at the harmonics *nf*_0_ (<~ 100 GHz). These acoustic spectral “lines” will be Lorentzian in shape, due to the exponentially decaying intensity of the light pulses in the train. Their full width at half maximum (FWHM) intensity is determined by the characteristic decay time. For the cavity we used, the product of the mirror reflectances was measured as ~ 0.95, giving a characteristic decay time of ~ 20 *t*_0_. Therefore, with the cavity set for a frequency of 40 GHz, we would expect a spectral width of order a GHz.

To characterize the pulses produced using this setup we used a conventional femtosecond optical pump-probe picosecond acoustic measurement: a train of 800 nm-wavelength pump light pulses was applied directly on the surface of a 17 micron-thick wafer of GaAs. The generated acoustic signal was probed on the opposite surface using a delayed optical probe pulse, derived from the same laser beam and frequency doubled to 400 nm-wavelength. The reflectance of the GaAs is modulated by the acoustic strain, and so a measurement of the intensity of the reflected probe pulse as a function of the time delay gives the temporal evolution of the acoustic signal. Figure S2(a) shows the temporal acoustic signal with the cavity set for *f*_0_ = 40 GHz. The individual strain pulses in the train are clearly resolved. The spectrum of the acoustic signal obtained by taking the FFT of the temporal signal in Fig. S2(a) is shown in Fig. S2(b). Clear peaks are seen at *f*_0_ = 40 GHz, and at 2*f*_0_ = 80 GHz.

This result shows that we are not only able to generate the quasi-monochromatic acoustic signal using this arrangement, but the acoustic signal is also able to propagate through at least 17 microns of GaAs at room temperature and be detected at the other side.

**B. Theory of acousto-electric mixing in Schottky diode**

As discussed in the main part of the paper, we consider mixing process caused by the non-linearity of the Schottky diode current-voltage characteristic. We consider the circuit shown in Fig. 5 of the paper. The capacitance of the diode (~10 fF) is low enough so that we can neglect the corresponding reactive component of the diode current. As a result, the diode current, $I_{d}$, consists from the following two contributions:

$I_{d}=I_{0}+I_{ac}$. (1)

Here $I_{0}(U_{d})$, $U_{d}$ being the diode voltage, is the active diode current caused by electron transfer through the Schottky barrier, which determines the diode dc current-voltage characteristics, and $I_{ac}$ is the current driven by the strain pulse due to screening of the strain-induced deformation potential in the metal. In general, $I_{ac}$ is a complicated function of time, but if the strain evolution is slower than characteristic time $l_{s}/v_{s}$, where $l_{s}$ is the screening length and $v_{s}$ is the sound velocity, then $I_{ac}=\frac{C_{d}E_{1}}{e}\frac{du_{zz}}{dt}$, where $E_{1}$ is the effective deformation potential in the metal, $e$ is the elementary charge, $C_{d}$ is the diode capacitance and $u_{zz}$ is strain in the metal near the interface with the semiconductor. In the following, we assume the finite-value LO voltage, while the strain-caused $I_{ac}$ is treated as a perturbation. In this case the zero-approach diode bias, $U_{d}^{(0)}$, is determined by the solution of the algebraic equation

$U_{0}+V_{LO}\cos(\omega_{LO}t-\phi)=U_{d}^{(0)}+R_{LO}I_{0}(U_{d}^{(0)})$. (2)

Here we take into account that, for signal averaging purposes, the measurements are repeated many times with the phase of the LO signal, $\phi$, changing randomly from measurement to measurement.

Under the above mentioned conditions, the acoustic-induced perturbation of the bias at the load resistor $R$ is given by

$U^{(1)}=\frac{I_{ac}R}{1+(R+R_{LO})I'_{0}(t)}$, (3)

where $I'_{0}$ is the derivative of the diode characteristics $I_{0}$ with respect to voltage calculated for $U_{d}=U_{d}^{(0)}$. Naturally, $U^{(1)}$ is a periodic function with the period $2\pi/\omega_{\text{LO}}$.

In the following we denote $\frac{du_{zz}}{dt}\equiv g(t)$ and define the Fourier components by tilde variables, e.g. $\tilde{g}(\omega)\equiv(2\pi)^{-1/2}\int_{-\infty}^{\infty} g(t)\exp(i\omega t)dt$. In our case the acoustic signal is a train of contributions attenuated and delayed due to reflection of the optical pump pulses in the Fabry-Perot cavity: $g(t)=\sum_{n=0}^{\infty} (r_{1}r_{2})^{n}g_{0}(t-nt_{0})$, where $r_{1,2}$ are the reflection coefficients of the optical mirrors composing the Fabry-Perot optical cavity, $t_{0}$ is the round-trip time of the light pulse within the cavity, and $g_{0}(t)$ describes the shape of the acoustic signal that is produced by the first member of the train series (*n* = 0). Such bouncing gives rise to peaks in the spectrum of $g(t)$ at the harmonics of $\omega_{0}\equiv2\pi/t_{0}$:

. (4)

For a high enough optical cavity quality (Q-) factor, the spectrum of $g(t)$ has narrow peaks at $\omega=\omega_{0}n$. Under the experimental conditions we have $2\omega_{0}\approx\omega_{LO}$. Therefore, the low-frequency spectrum of $U^{(1)}$ is determined by the first harmonic amplitudes of $\left(1+(R+R_{LO})I'_{0}(t)\right)^{-1}$. In the experiment the analyzed value is the IF power, $P$, which is defined as , where means averaging over $\phi$. Introducing the Fourier expansion $\left(E_{1}/e\right)RC_{d}\left(1+(R+R_{LO})I'_{0}(t)\right)^{-1}=A_{0}+\sum_{n=1}^{\infty} \left(A_{n}\cos n\omega_{LO}(t-t_{0})+B_{n}\sin n\omega_{LO}(t-t_{0})\right)$, we obtain

. (5)

For $r_{1}r_{2}$ close to unity, under the conditions of experiment with $\Delta\omega\equiv2\omega_{0}-\omega_{LO}<<\omega_{0}$, the spectrum of $P$ has Lorentz-like form:

, (6)

where $\gamma=\omega_{0}(1-r_{1}r_{2})(2\pi\sqrt{r_{1}r_{2}})^{-1}$. Note, that the results in the main part of the paper are presented assuming frequency $f=\omega/(2\pi)$ as an independent value; therefore, we rewrite (6) in the form

. (7)

where $\gamma_{f}=f_{0}(1-r_{1}r_{2})(2\pi\sqrt{r_{1}r_{2}})^{-1}$.

The theoretical results shown in the main paper were obtained assuming ideal diode current-voltage ($I_{0}$- $U_{d}$) characteristics $I_{0}(U_{d})=I_{th}(\exp(eU_{d}/(k_{B}T))-1)$, with $I_{th}=2\cdot10^{-14}\text{A}$, dc bias $U_{0}=0.5 V$, diode capacitance $C_{d}=10^{-14}F$, and LO amplitude $V_{LO}=0.6 V$. The strain pulse spectrum was modeled by the expression $\tilde{g}_{0}(\omega)=\sqrt{2/\pi}u_{zz}^{(\max)}\exp\left(-\omega^{2}\tau^{2}/2\right)$ with strain amplitude $u_{zz}^{(\max)}=10^{-3}$ and $\tau=2 ps$, $r_{1,2}=0.95$, and the deformation potential constant $E_{1}=6 \text{eV}$ was used.

**C. Video of IF spectrum as a function of acoustic signal frequency**

The “radio tuning bar” top shows the frequency of the acoustic signal, changing as the Fabry-Perot cavity spacing is changed. The local oscillator (LO) frequency is at the centre (94 GHz). The graph to the right shows the intermediate frequency (IF) signal recorded on the oscilloscope.

The small peak which remains at ~ 2 GHz when the IF moves to higher frequencies is an artefact due to “ringing” of a parasitic resonant circuit associated with the Schottky diode mounting.


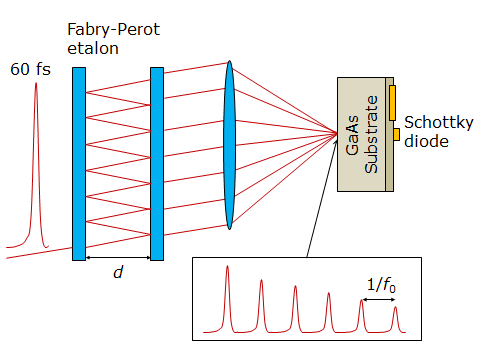


Figure S1. Fabry-Perot etalon pulse shaper: a single femtosecond laser pulse is input and results in a train of output pulses with exponentially decaying intensity. These are focussed onto the surface of the GaAs substrate opposite to the Schottky diode. For clarity the laser beam is shown as entering the cavity at an angle, but in practice, to get the best overlap of the pulses on the sample, the light enters the cavity in a direction exactly perpendicular to the plane of the mirrors.


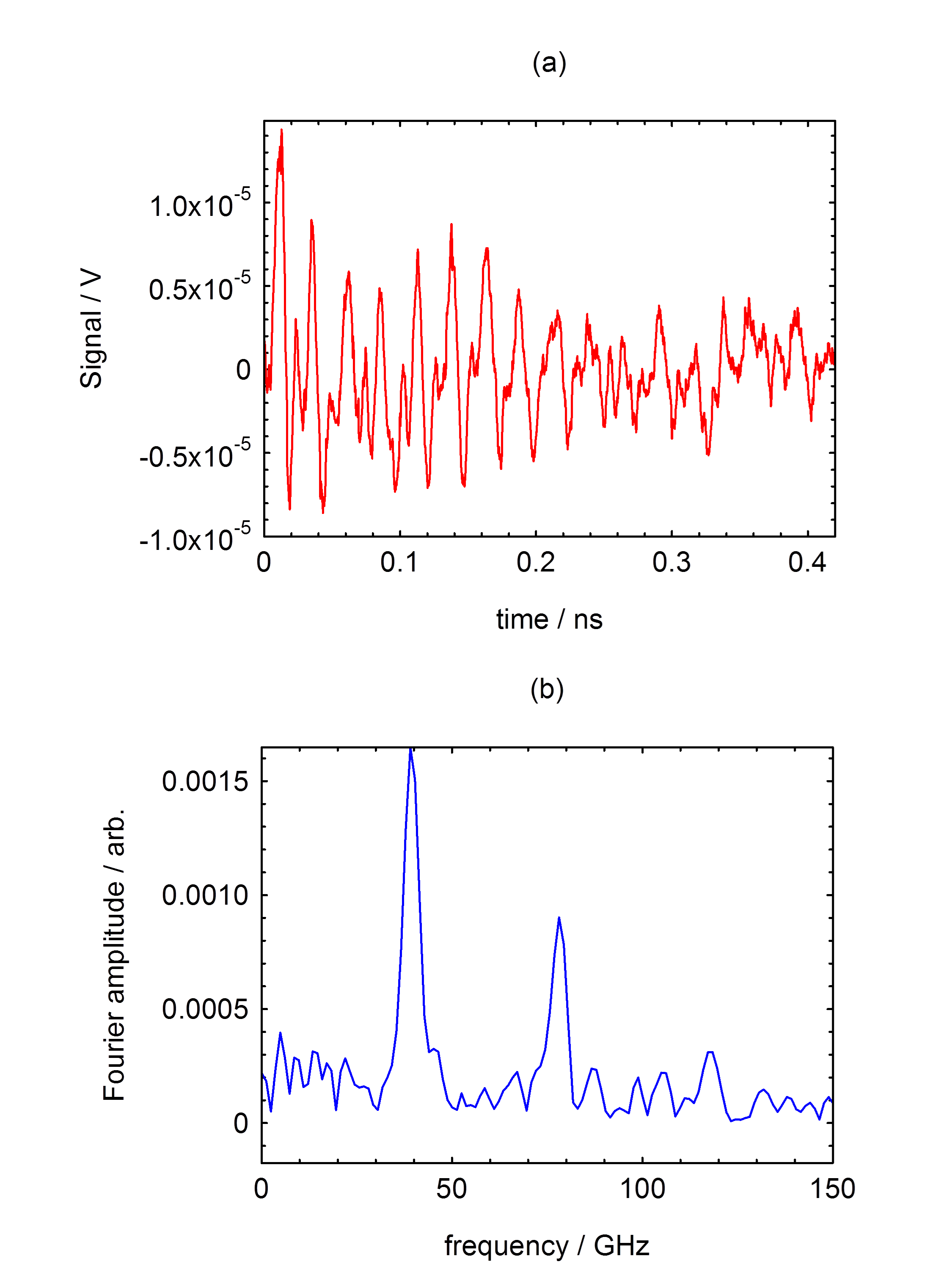


Figure S2. Characterisation of the acoustic wave packet: (a) temporal pump-probe trace with the cavity set for *f*_0_ *=* 40 GHz. Zero time corresponds to the acoustic packet, generated on one face of the GaAs wafer, reaching the opposite face where it is probed. About 17 individual acoustic strain pulses can be picked out; (b) Fourier spectrum of the acoustic signal showing peaks at the fundamental (*f*_0_ *=* 40 GHz) and at the harmonics 2*f*_0_ and 3*f*_0_.
